# Supplementary material for: Machine Learning Accelerates Crystallization for Structure Determination
Source: Angew Chem Int Ed Engl. 2026 May 4;65(25):e1218503. doi: 10.1002/anie.1218503 (PMC13266958; doi:10.1002/anie.1218503)

checkCIF/PLATON report

Structure factors have been supplied for datablock(s) lcz046

THIS REPORT IS FOR GUIDANCE ONLY. IF USED AS PART OF A REVIEW PROCEDURE FOR PUBLICATION, IT SHOULD NOT REPLACE THE EXPERTISE OF AN EXPERIENCED CRYSTALLOGRAPHIC REFEREE.

No syntax errors found.   CIF dictionary   Interpreting this report

Datablock: lcz046

Bond precision: C-C = 0.0074 A   Wavelength=1.54184

Cell:	a=8.1897(2)	b=12.9426(3)	c=17.1971(4)
	alpha=90.273(2)	beta=97.670(2)	gamma=103.305(2)
Temperature:	100 K		

	Calculated	Reported
Volume	1756.84(7)	1756.84(7)
Space group	P -1	P -1
Hall group	-P 1	-P 1
Moiety formula	C28 H19 Ag3 F18 N6 O2	C15 H3 Ag3 F18 N6, C13 H16 O2
Sum formula	C28 H19 Ag3 F18 N6 O2	C28 H19 Ag3 F18 N6 O2
Mr	1137.10	1137.10
Dx,g cm-3	2.150	2.150
Z	2	2
Mu (mm-1)	14.542	14.542
F000	1096.0	1096.0
F000'	1101.21	
h,k,lmax	10,16,21	10,16,21
Nref	7575	7198
Tmin,Tmax	0.116,0.151	0.603,1.000
Tmin'	0.019	

Correction method= # Reported T Limits: Tmin=0.603 Tmax=1.000
AbsCorr = MULTI-SCAN

Data completeness= 0.950 Theta(max)= 78.592

R(reflections)= 0.0437(6571) wR2(reflections)= 0.1206(7198)

S = 1.056 Npar= 518

The following ALERTS were generated. Each ALERT has the format
test-name_ALERT_alert-type_alert-level.
Click on the hyperlinks for more details of the test.

Alert level C

```
CRYSC01_ALERT_1_C The word below has not been recognised as a standard
            identifier.
            dull
PLAT042_ALERT_1_C Calc. and Reported MoietyFormula Strings  Differ     Please Check
              Calc: C28 H19 Ag3 F18 N6 O2
              Rep.: C15 H3 Ag3 F18 N6, C13 H16 O2
PLAT213_ALERT_2_C Atom F00Q            has ADP max/min Ratio .....        3.2 prolat
PLAT213_ALERT_2_C Atom F00R            has ADP max/min Ratio .....        3.6 prolat
PLAT309_ALERT_2_C Single Bonded Oxygen (C-O > 1.3 Ang) ..........        O00N Check
PLAT362_ALERT_2_C Short  C(sp3)-C(sp2) Bond  C01F     - C01H     .       1.38 Ang.
PLAT906_ALERT_3_C Large K Value in the Analysis of Variance ......      2.734 Check
PLAT911_ALERT_3_C Missing FCF Refl Between Thmin & STh/L=    0.600         30 Report
               -2  2  0,  -1  2  0,  -2-14  1,   0 -1  1,   1 -1  1,  -1  1  1,
                0  1  1,  -6 -5  2,  -8 -3  2,  -8 -2  2,  -6  0  2,   0-14  8,
                1-14  8,  -1-10 15,  -3 -9 15,  -2 -9 15,  -1 -9 15,  -2 -8 15,
               -1 -8 15,  -2 -9 16,  -1 -9 16,  -3 -8 16,  -2 -8 16,  -1 -8 16,
               -2 -8 17,  -1 -8 17,  -3 -7 17,  -2 -7 17,  -2 -7 18,  -3 -6 18,
PLAT934_ALERT_3_C Number of (Iobs-Icalc)/Sigma(W) > 10 Outliers ..          1 Check
                3-14  6,
PLAT977_ALERT_2_C Check Negative Difference Density on H01I      .      -0.57 eA-3
PLAT977_ALERT_2_C Check Negative Difference Density on H01J      .      -1.00 eA-3
```

Alert level G

```
PLAT083_ALERT_2_G SHELXL Second Parameter in WGHT  Unusually Large      10.94 Why ?
PLAT154_ALERT_1_G The s.u.'s on the Cell Angles are Equal ..(Note)      0.002 Degree
PLAT242_ALERT_2_G Low    'MainMol' Ueq as Compared to Neighbors of       C016 Check
PLAT242_ALERT_2_G Low    'MainMol' Ueq as Compared to Neighbors of       C019 Check
PLAT242_ALERT_2_G Low    'MainMol' Ueq as Compared to Neighbors of       C01A Check
PLAT242_ALERT_2_G Low    'MainMol' Ueq as Compared to Neighbors of       C01D Check
PLAT242_ALERT_2_G Low    'MainMol' Ueq as Compared to Neighbors of       C01K Check
PLAT720_ALERT_4_G Number of Unusual/Non-Standard Labels ..........         76 Note
              Ag01    Ag02    Ag03    F004    F005    F006    F007    F008
              F009    F00A    O00B    N00C    N00D    N00E    F00F    F00G
              N00H    F00I    N00J    N00K    F00L    F00M    O00N    F00O
              F00P    F00Q    F00R    F00S    C00T    H00T    F00U    C00V
              H00V    C00W    C00X    C00Y    H00Y    C00Z    C010    C011
              C012    C013    C014    C015    C016    C017    C018    C019
              C01A    C01B    H01A    H01B    H01C    C01C    H01D    C01D
              C01E    H01E    C01F    C01G    H01F    H01G    H01H    C01H
              H01I    H01J    C01I    C01J    H01K    H01L    H01M    C01K
              C01L    H01N    H01O    H01P
```

```
PLAT883_ALERT_1_G Absent Datum for _atom_sites_solution_primary ..     Please Do !
PLAT910_ALERT_3_G Missing FCF Reflection(s) Below Theta(Min)[Deg]=       3.51 Note
                0  0  1,
PLAT912_ALERT_4_G Missing # of FCF Reflections Above STh/L=  0.600        346 Note
PLAT913_ALERT_3_G Missing # of Very Strong Reflections in FCF ....          1 Note
               -2  2  0,
PLAT941_ALERT_3_G Average HKL Measurement Multiplicity ..........         2.4 Low
PLAT969_ALERT_5_G The 'Henn et al.' R-Factor-gap value ..........       3.163 Note
              Predicted wR2: Based on SigI**2  3.81 or SHELX Weight 11.42
PLAT978_ALERT_2_G Number C-C Bonds with Positive Residual Density.          0 Info
```

```
   0 ALERT level A = Most likely a serious problem - resolve or explain
   0 ALERT level B = A potentially serious problem, consider carefully
  11 ALERT level C = Check. Ensure it is not caused by an omission or oversight
  15 ALERT level G = General information/check it is not something unexpected

   4 ALERT type 1 CIF construction/syntax error, inconsistent or missing data
  13 ALERT type 2 Indicator that the structure model may be wrong or deficient
   6 ALERT type 3 Indicator that the structure quality may be low
   2 ALERT type 4 Improvement, methodology, query or suggestion
   1 ALERT type 5 Informative message, check
```

Validation response form

Please find below a validation response form (VRF) that can be filled in and pasted into your CIF.

```
# start Validation Reply Form
_vrf_CRYSC01_lcz046
;
PROBLEM: The word below has not been recognised as a standard
RESPONSE: ...
;
_vrf_PLAT042_lcz046
;
PROBLEM: Calc. and Reported MoietyFormula Strings  Differ     Please Check
RESPONSE: ...
;
_vrf_PLAT213_lcz046
;
PROBLEM: Atom F00Q            has ADP max/min Ratio .....        3.2 prolat
RESPONSE: ...
;
_vrf_PLAT309_lcz046
;
PROBLEM: Single Bonded Oxygen (C-O > 1.3 Ang) ..........       O00N Check
RESPONSE: ...
;
_vrf_PLAT362_lcz046
;
PROBLEM: Short  C(sp3)-C(sp2) Bond  C01F     - C01H     .       1.38 Ang.
RESPONSE: ...
;
_vrf_PLAT906_lcz046
;
PROBLEM: Large K Value in the Analysis of Variance ......      2.734 Check
```

```
RESPONSE: ...
;
_vrf_PLAT911_lcz046
;
PROBLEM: Missing FCF Refl Between Thmin & STh/L=    0.600         30 Report
RESPONSE: ...
;
_vrf_PLAT934_lcz046
;
PROBLEM: Number of (Iobs-Icalc)/Sigma(W) > 10 Outliers ..          1 Check
RESPONSE: ...
;
_vrf_PLAT977_lcz046
;
PROBLEM: Check Negative Difference Density on H01I      .      -0.57 eA-3
RESPONSE: ...
;
# end Validation Reply Form
```

It is advisable to attempt to resolve as many as possible of the alerts in all categories. Often the minor alerts point to easily fixed oversights, errors and omissions in your CIF or refinement strategy, so attention to these fine details can be worthwhile. It is up to the individual to critically assess their own results and, if necessary, seek expert advice.

PLATON version of 17/09/2025; check.def file version of 17/09/2025

---

duplicate check

No duplication found

Datablock lcz046 - ellipsoid plot

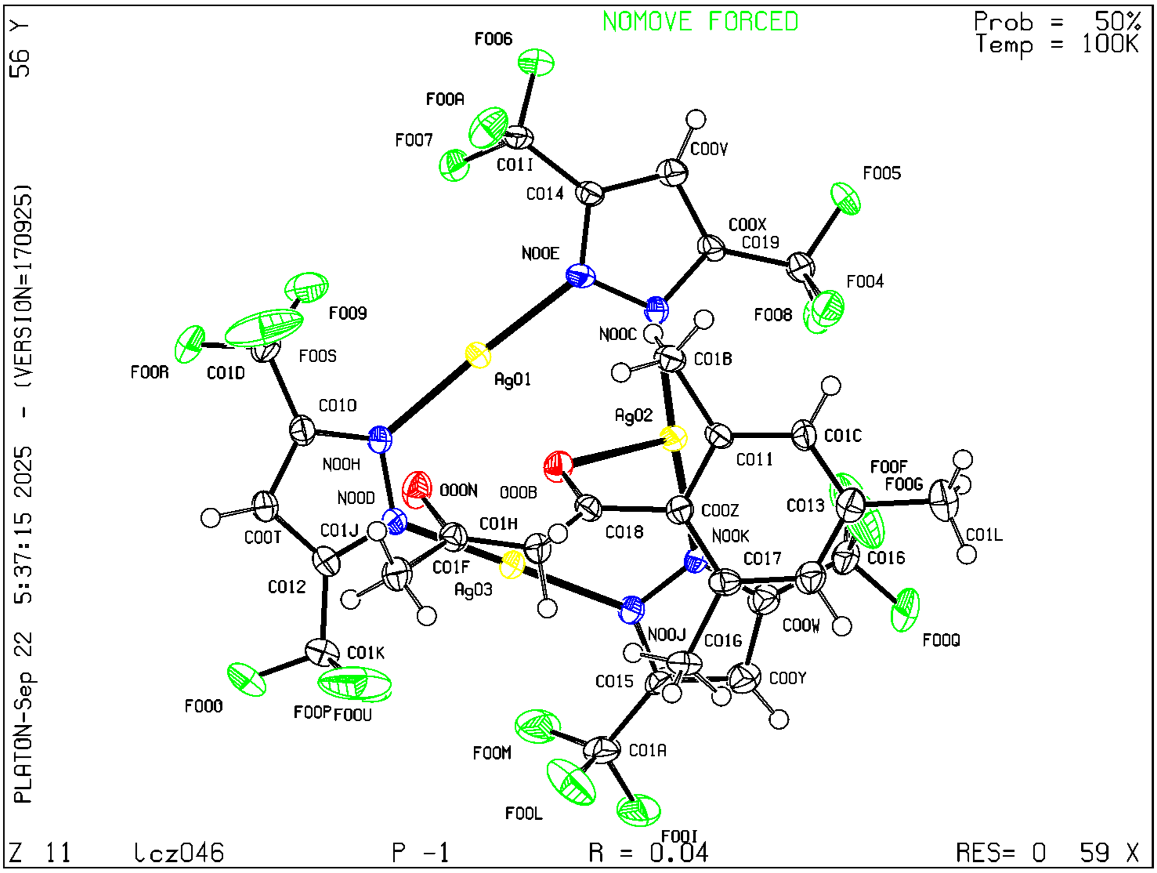

Supplement: Supplementary file 2 — Supporting File 2: anie72462‐sup‐0002‐DataFile.zip. [file ANIE-65-e1218503-s002.zip › supplementary_materials_crystallographic_data submit.zip!/cif_and_checkcif(1-114)/Ag3Pz3_46/Ag3Pz3_46_checkcif.pdf]
